# Three-Dimensional Human Pose Estimation from Sparse IMUs through Temporal Encoder and Regression Decoder

**DOI:** 10.3390/s23073547

**Published:** 2023-03-28

**Authors:** Xianhua Liao, Jiayan Dong, Kangkang Song, Jiangjian Xiao

**Affiliations:** 1 School of Information Science and Engineering, Ningbo University, Ningbo 315211, China; 2 Ningbo Institute of Materials Technology and Engineering, Chinese Academy of Sciences, Ningbo 315201, China

**Keywords:** three-dimensional human pose, sparse IMUs, encoder–decoder, temporal convolutional encoder, human kinematics hierarchy, regression decoder

## Abstract

Three-dimensional (3D) pose estimation has been widely used in many three-dimensional human motion analysis applications, where inertia-based path estimation is gradually being adopted. Systems based on commercial inertial measurement units (IMUs) usually rely on dense and complex wearable sensors and time-consuming calibration, causing intrusions to the subject and hindering free body movement. The sparse IMUs-based method has drawn research attention recently. Existing sparse IMUs-based three-dimensional pose estimation methods use neural networks to obtain human poses from temporal feature information. However, these methods still suffer from issues, such as body shaking, body tilt, and movement ambiguity. This paper presents an approach to improve three-dimensional human pose estimation by fusing temporal and spatial features. Based on a multistage encoder–decoder network, a temporal convolutional encoder and human kinematics regression decoder were designed. The final three-dimensional pose was predicted from the temporal feature information and human kinematic feature information. Extensive experiments were conducted on two benchmark datasets for three-dimensional human pose estimation. Compared to state-of-the-art methods, the mean per joint position error was decreased by 13.6% and 19.4% on the total capture and DIP-IMU datasets, respectively. The quantitative comparison demonstrates that the proposed temporal information and human kinematic topology can improve pose accuracy.

## 1. Introduction

In computer vision, games, sports, medicine, virtual reality, augmented reality, and other fields, three-dimensional (3D) human pose reconstruction has long been a challenging problem. However, owing to the diversity of human poses, predicting complex human actions remains a challenge. Many previous works have proposed image-based approaches where the pose is recovered by analyzing image data, which can consist of multi-view [1,2,3], RGB-D [4,5], video [6,7], or single-view images [8,9]. However, state-of-the-art image-based methods of three-dimensional human pose estimation are still sensitive to occlusion. Furthermore, many methods have limitations that prevent their use in situations involving outdoor spaces or in indoor scenarios spanning multiple rooms. Especially in outdoor scenes, they produce significantly erroneous predictions, even when most parts of the human body are observable.

Recently, human body researchers have begun investigating alternative data, such as inertial measurement units (IMUs). IMUs can effectively solve the viewpoint limitation of accessible optical sensors and produce the rotation information of joint points. The IMU-based systems focus on wearable sensor estimation of body pose by binding some inertial sensing peripherals to key joints of the human body and capturing the direction and acceleration of these joints. However, the commercial IMU-based system usually relies on dense and complex wearable sensors and time-consuming calibration, e.g., 17 nodes employed by the Xsens Animate suit, which are invasive to the subject and hinder the free movement of the body. With the development of the parametric statistical human body shape models, a realistic and controllable three-dimensional mesh of the human body can be generated from only a few parameters, e.g., shape parameters and relative rotations of body parts. Some recent studies [10,11,12,13,14] have proposed using only six IMUs to solve based-model human pose estimation and adopted deep neural network (DNN)-based algorithms.

**Figure 1:**
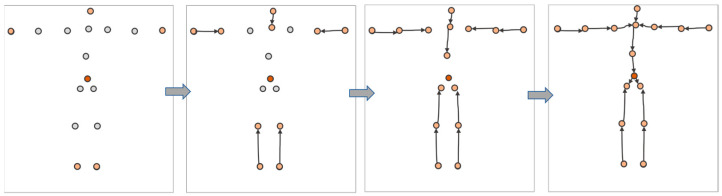
Illustration of hierarchical regression based on kinematic tree. The process of sequentially generating the joint pose parameters is shown as white becomes a warm color.

**Figure 2:**
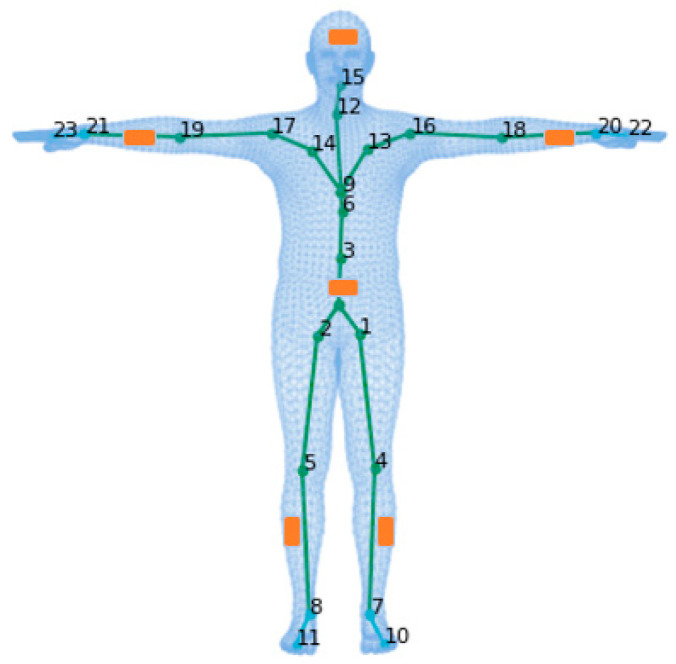
Illustration of the mesh, skeleton, and joints with the T-pose template. The line plot is a common kinematic representation of the human body by 24 key points. The triangle mesh is the skinned and vertex-based three-dimensional prior model in the SMPL. The placement of six IMUs in this study is indicated in orange.

However, the existing DNN-based algorithms fail to distinguish between poses on similar IMU measurement values, such as standing and sitting. Furthermore, the accuracy remains limited, and problems such as jitter occur during fast motion. To address these problems, we adopted the parametric three-dimensional human body model, skinned multi-person linear (SMPL) [15], with prior knowledge as an intermediate tool and jointly modeled two intrinsic relationships. (1) Temporal relationship: given a specific motion trajectory of the measurement data, when action ambiguity occurs, the temporal relationship is used to infer the pose of the current frame from the surrounding information. (2) The dependence among joints: the human kinematics tree preserves prior knowledge, and our method iteratively generates pose parameters, shown in Figure 1. The parametric three-dimensional human body model SMPL with a hierarchical structure composed of 24 joints, which preserves the parent relations of the joints, as shown in Figure 2. Based on the above relationship, this paper proposes a multistage encoder–decoder network combining a temporal convolutional encoder and a human kinematics regression decoder.

Different from the previous temporal-based methods, we adopted the temporal encoder and kinematic encoder strategy to reconstruct the three-dimensional human pose. The temporal convolutional encoder comprises several cascaded blocks, each composed of the temporal convolutional layer and bidirectional recurrent neural network (biRNN) [16] with a long short-term memory (LSTM) [17] unit. The temporal convolution layer implements one-dimensional (1D) convolution in the time dimension. It aggregates the data from adjacent frames into a single frame, increasing the temporal receptive field while reducing the number of calculations. Then, the biRNN aggregates the temporal cues in the context, enhancing feature information from the current frame and eliminating ambiguity. Some methods [10,11,12,13,14] use multilayer perceptron (MLP) layers or other iterative linear regression methods [6,7,8,9] to generate body pose parameters, where all joint pose parameters were generated simultaneously. However, these methods did not consider the interdependence of human joints. To exploit the dependence among joints, we adopted a kinematic regression decoder that assigns each joint a spatial attention layer and linear regressor. The regression generates the pose parameters for that joint. To estimate joint pose parameters, besides the IMU feature, we took the predicted pose parameters of its descendants as the input of the linear regressor. The bottom-to-top regression method enables the model to capture the internal relationship between joints effectively and reduce the prediction error.

To evaluate the effectiveness of the proposed approach, we conducted experiments on the Total Capture [18,19,20] and deep inertial poser (DIP)-IMU [11] datasets, which are commonly used benchmarks for human pose estimation. Compared with the state-of-the-art sparse-IMU-based method proposed by Puchert et al. [14], the three-dimensional joint position error was decreased by 13.6% on the Total Capture dataset and 19.4% on the DIP-IMU dataset. The experimental results demonstrate the effectiveness of encoding temporal features and decoding spatial features.

The main contributions of this paper can be summarized as follows.

1. This paper proposes a method based on an encoder–decoder framework that encodes temporal features and decodes spatial features to generate the three-dimensional human pose, which can alleviate the ambiguity of the conventional methods.
2. A spatial-attention and regression network is proposed to enhance the local spatial features of inertial measurements and relies on human topological regression to pay attention to the features of joints.
3. The method achieves state-of-the-art performance on two benchmarks for model-based three-dimensional human pose estimation, providing a solution for three-dimensional human motion capture in some unrestricted environments in practice.

## 2. Related Work

Many works focus on human pose estimation, using approaches that can be mainly divided into optical, inertial, and hybrid methods. As our method only requires IMU measurements as input, we do not discuss purely image-based approaches. Here, we focus on image-IMU-based and IMU-based approaches.

### 2.1. Image-IMU-Based Methods

As image-based pose estimation [6,7,8,9] suffers from both external and self-occlusions, fusing images and IMUs to achieve more robust pose estimation [21,22,23,24,25], which has recently garnered considerable attention. One category of approaches [18,25] estimated three-dimensional human poses by minimizing the energy function related to the IMU image features. Another category utilized a two-stream network to concatenate the pose embeddings obtained from images and IMUs to regress the final pose. Trumble et al. [19,21] proposed feature-based pose estimation to regress human poses from combined features obtained from images and IMUs. Zhang et al. [25] integrated multi-view images and IMUs. The rotation information was fused with image features at an early stage to improve two-dimensional pose estimation directly. In the stage of three-dimensional pose estimation, IMU data were used to optimize the results through three-dimensional geometry optimization. Although the two-stage method can achieve state-of-the-art performance using images alone, it has serious limitations and only works well in indoor scenes with multi-view cameras. The above methods have significant limitations in severe occlusions and require the human body to be in the camera view with a limited range of motion. Our approach does not require the input of visual data and thus does not suffer from these limitations.

### 2.2. IMU-Based Methods

IMU-based pose estimation is not limited to occlusions and activity spaces. With the development of micro-electromechanical systems, IMUs that measure acceleration and direction have garnered increasing attention. Some methods have been proposed using only IMUs rather than a marker-based system to restore three-dimensional human postures, such as the commercial inertial motion capture systems [26] using 17 wearable IMUs to obtain fully the orientations of all bones of the moving body model. However, deploying many sensors is seriously invasive to the subject and hinders the free movement of the subject. In addition, the calibration of multiple sensors often takes a long time. Therefore, reducing the number of IMUs is desirable [27,28]. However, motion capture based on sparse IMUs is ambiguous and challenging. In the methods proposed by Slyper et al. [27] and Tautges et al. [28], data from five accelerometers and poses were retrieved from a pre-established motion database. The pioneering work on the sparse inertial poser (SIP) [10] proposed to solve human pose estimation using only six IMUs, which is an iterative optimization method and requires access to the entire motion sequence. The DIP [11] was the first to employ a deep learning method that used a biRNN [16] and provided the DIP-IMU dataset. Yi et al. [12,13] proposed multi-stage task completion to estimate key-point position information before regressing joint rotation information as an intermediate result to connect IMU measurements into the next stage of the network, significantly improving the accuracy and reducing the running time. Nevertheless, the third stage of their transpose [12] network is an inverse kinematics (IK) solver, which would produce key-point ambiguity. The IK mathematical process finds the positions of body joints to create relative rotations. Furthermore, simply using the IMU as input to estimate the joint rotation does not introduce any prior knowledge about the human body. Puchert et al. [14] proposed converting the parent–child relationship between human joints into a graph structure and adding the graph structure to the LSTM [17] network to achieve pose estimation.

This study was the first to apply a temporal feature encoder and kinematics hierarchy-based decoder to sparse IMU-based three-dimensional human pose estimation. In contrast to previous work, the current mainstream deep learning methods based on sparse IMU pose estimation still model temporal features. As the IK problem is ill-posed, previous works were proposed, which can only be eliminated according to motion history.

However, they disregard the topology of the human body. Therefore, our proposed method models the temporal and human joint spatial structure relationships. Our experiments demonstrated that simultaneously modeling both relationships can achieve superior results compared to those obtained by modeling only the temporal relationship.

## 3. Methods

This section first overviews the parametric three-dimensional human body model (SMPL) [15]. Secondly, it introduces the proposed approach based on the encoder–decoder framework. Finally, it describes the IMU feature extractor, temporal encoder, and kinematic regression decoder in detail.

### 3.1. SMPL

SMPL [15] is a skinned and vertex-based three-dimensional prior model of the human body learned from thousands of three-dimensional body scans. The human skeleton is a hierarchy of 24 joints defined by a kinematic tree, which preserves the parenting of the joints. The SMPL is parameterized by $\theta \in {\mathbb{R}}^{72}$ and $\beta \in {\mathbb{R}}^{10}$, where θ represents the rotation of the corresponding 23 joints relative to the parent joint and one root (pelvic) global orientation, and β is a human morphological vector composed of 10 scalars. Each scalar indicates that the human body expands or contracts in a specific direction. A shape blended T-pose of the SMPL is shown in Figure 2. The body mesh $M\in {\mathbb{R}}^{N\times 3}$ can be obtained from M(β,θ) where N=6890. According to previous research, the position of IMU binding is provided in Figure 2, as indicated in orange. IMUs are bound sequentially to the left lower wrist, right lower wrist, left lower leg, right lower leg, head, and pelvis. Except for the IMU at the root (pelvic) position, the others are collectively referred to as the leaf-joint IMUs. The leaf joint inertial measurements are aligned with the root joint:

(1) $$A_{leaf}={\bar{O}}_{leaf}^{-1}({\bar{A}}_{leaf}-{\bar{A}}_{root}),$$

(2) $$O_{leaf}={\bar{O}}_{root}{\bar{O}}_{leaf},$$

where $O\in {\mathbb{R}}^{3\times 3}$ represents the orientation and $A\in {\mathbb{R}}^{3}$ indicates the acceleration. $A_{leaf},O_{leaf}$ are the input values of our network.

### 3.2. Framework Overview

**Figure 3:**
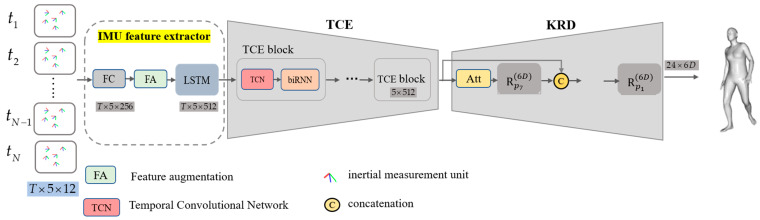
Overview of our proposed framework for human pose estimation from sparse IMUs. Our network framework is divided into three parts. The first part linearly encodes the IMU measurement data and combines the difference between two consecutive frames with the position feature to enhance the feature. The second part is the temporal encoder, which extracts feature information from past and future frames and contains several cascaded blocks. The third part is the kinematic regression decoder, which generates the pose parameters using hierarchical regression defined by the human motion tree.

Figure 3 shows the proposed network framework. The network receives an input sequence of length T×N×Fin, where N=5 denotes the number of leaf-joint IMUs and Fin=12. It includes a 3×3 rotation matrix and three-dimensional acceleration. Following the IMU sensor setting of the DIP [11], the orientation and acceleration data of each sensor are firstly transformed to the global inertial coordinates and then converted into the SMPL global coordinates. In contrast with previous research, the input data are not flattened to extract the feature information of human spatial relationships. The network firstly encodes a vector T×N×512 through the IMU feature extractor. The vector is then used as input into a temporal encoder, which models these basic temporal features and outputs an N×512 vector. Finally, our proposed kinematic regression decoder is employed to estimate pose parameter θ. Here, following Transpose [12], we use the six-dimensional (6D) rotation representation for faster convergence.

These parameters can utilize the SMPL to compute three-dimensional key points, $\hat{X}(\theta )=WM(\theta ,\beta )$, where parameters β are constants, $\hat{X}(\theta )\in {\mathbb{R}}^{24\times 3}$, and W is a linear regressor. The body mesh $\hat{V}(\theta )=M(\beta ,\theta )$, where $\hat{V}(\theta )\in {\mathbb{R}}^{6890\times 3}$. Therefore, the constraints of the network employed in this study include the mesh, three-dimensional key points, and pose parameters θ. The loss function in this study is defined as

(3) $$L_{all}=\tau L_{vertices}+\varphi L_{keypoints}+\omega L_{SMPL},$$

where $L_{vertices}$ denotes the body mesh loss, $L_{keypoints}$ denotes the three-dimensional key-point loss, and $L_{SMPL}$ denotes the pose parameter loss. τ,φ, and ω denote the corresponding weights of the three loss items. These losses are denoted as follows:

(4) $$L_{vertices}=|\;|V-\hat{V}|\;{|}_{1},$$

(5) $$L_{keypoints}=|\;|X-\hat{X}|\;{|}_{2},$$

(6) $$L_{SMPL}=|\;|\theta -\hat{\theta }|\;{|}_{2},$$

where $\theta \in {\mathbb{R}}^{24\times 3\times 3}$, and V,X, and θ represent the ground truths of $\hat{V},\hat{X}$, and $\hat{\theta }$, respectively.

### 3.3. IMU Feature Extractor

Following the IMU measurement processing of Transpose [12], our experiment demonstrates that the network converges faster when the acceleration is scaled to 30 times the original value. The extractor firstly encodes a T×N×5 vector through a fully connected layer as the position feature, which can only represent the spatial feature. The difference between two consecutive frames is combined with the position feature to enhance the features and improve the capture of temporal features. The feature augmentation process is as follows:

(7) $${f^{\prime }}_{previous}(t)=f(t)-f\left(t-1\right),$$

(8) $$f_{augmentation}(t)=Concat(f(t)-{f^{\prime }}_{previous}(t)),$$

where Concat(⋅) indicates the concatenation operation, inspired by Si et al. [29].

After one layer of the LSTM [17] network, the joint features are smoothed. Therefore, the features of the current frame include spatial location features and temporal features, and the preprocessing outputs a matrix of size T×N×512. Then, the vector is used as input to a temporal encoder.

### 3.4. Temporal Convolutional Encoder

**Figure 4:**
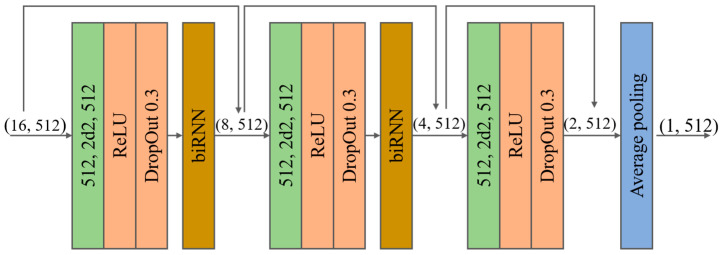
Overview of the temporal convolutional encoder. The output has 16 frames, and the feature dimension has 512. The convolutional layer is shown in green, where (512, 2d2, and 512) represents 512 input channels, a convolution kernel size of two, and a stride of 2512 output channels. Finally, average pooling produces the feature vector from a single frame.

The temporal encoder, which benefits from long-term modeling using temporal data, extracts useful feature information from previous and future frames. Figure 3 shows the temporal encoder, which comprises several cascaded blocks containing a temporal convolutional network (TCN) and biRNN [16]. The TCN performs a one-dimensional convolution in the time dimension. The convolution kernel and stride are both two, and the data from adjacent frames are combined into a single frame to increase the temporal receptive field and reduce the computation. The biRNN uses two LSTM layers to generate latent features for each input frame. Finally, average pooling is performed in the time dimension to output the feature information from a single frame after multiple frames of feature information have been output. The specific implementation is illustrated in Figure 4.

### 3.5. Kinematic Regression Decoder

**Figure 5:**
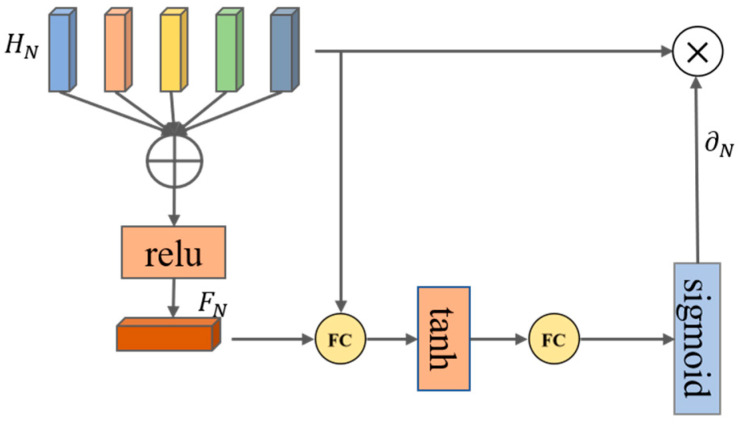
Illustration of the spatial attention network. We firstly aggregated the vector $H_{N}\in {\mathbb{R}}^{512\times 5}$ of all IMUs as a query feature $F_{N}\in {\mathbb{R}}^{512}$, then calculated the attention scores of all IMUs $\partial_{N}\in {\mathbb{R}}^{5}$. We used the non-linear function of Sigmoid due to the possibility of multiple key joints. The output of this network is a 5×512 -dimensional vector.

As demonstrated in Figure 2, the pose of the human body is controlled by 23 joints and a root joint, which are organized as a kinematic tree. The decoder assigns the spatial attention network and the linear regressor to 15 important joints appearing in green in Figure 2, and the attention network is employed to focus adaptively on the joint points related to the key point. The input of the spatial attention network is the IMU feature, which is output by the temporal encoder and contains rich spatial structural information and temporal dynamics that are beneficial in guiding the selection of key joints. The attention network enhances the hidden state and generates local features of the IMU associated with the key joints. An illustration of the spatial attention network is shown in Figure 5.

The attention scores of the five IMUs are determined as follows:

(9) FN=ReLU(∑iNHi),

(10) $$\partial_{N}=Sigmoid(U_{s}\tanh(W_{h}H_{N}+W_{f}F_{N}+b_{s})+b_{u}),$$

where $\partial_{N}=\{\partial_{1},\partial_{2},\partial_{3},\partial_{4},\partial_{5}\}$, $U_{s},W_{h},W_{f}$ are learnable parameter matrices, and $b_{u},b_{s}$ are biases, inspired by previous work [29].

In addition to the IMU local features output by the spatial attention network, we took the predicted pose parameters of its descendants as inputs into the linear regressor. According to the hierarchical relationship defined by the motion tree, the position of the joint is affected by its own pose parameters and those of its descendants. The more sub-joints, the greater the impact on the accuracy of the overall joint position estimation. Here, we established a dependency between parent and child joints, which is consistent with the kinematic tree structure. Then, we iteratively generated the pose parameter for each joint in hierarchical order according to the structure of kinematic tree. C(k) is the ordered set of the descendants of joint k, e.g., $C(1)=\left\{\left.10,7,4\right\}\right.$ in Figure 2. We took joints $\left\{\left.10,7,4,1\right\}\right.$ as an example. Following the previous work [11,12,13,14], the eight leaf joints appearing in cyan in Figure 2, which pose parameters are set to identity. We firstly predicted the pose parameters of joint 4, using the output feature ${\vec{x}}_{4}$ of the attention network and a learnable linear regressor, i.e.,

(11) $$R_{4}^{6D}=w_{4}concat({\vec{x}}_{4},R_{7}^{6D},R_{10}^{6D}),$$

for joint 1, the steps are as shown above, i.e.,

(12) $$R_{1}^{6D}=w_{1}concat({\vec{x}}_{1},R_{4}^{6D},R_{7}^{6D},R_{10}^{6D}),$$

where $w_{1},w_{4}$ are learnable parameter matrices.

This paper’s purpose is to benefit the joint hierarchical relationship provided by human kinematics, which is conducive to the generation of reasonable poses. For each joint, the bottom-top regression process causes the spatial attention network to focus more on the parent joint with more child joints.

### 3.6. Experimental Details

For the experiments with our model, we used Pytorch version 1.8, which is optimized with the Adam optimizer, and, after training with 200 epochs with an initial learning rate of 0.001, an exponential decay rate of 0.96 was observed, and a decay step of 2000 was observed. We simultaneously set up a GPU batch with a minimum size of 64 and two GPUs, and in all experiments, we used $\tau =1\times 10^{-2},\varphi =1$, and $w=2\times 10^{-1}$. The temporal encoder cascaded the dropout between blocks. The rotational information output by the model was a representation of six-dimensional degrees of freedom.

## 4. Experiments

### 4.1. Datasets and Metrics

#### 4.1.1. Data Preparation

According to previous research [11,12,13,14], our model was trained using synthetic data, generated from the Archive of Motion Capture as Surface Shapes (AMASS) motion capture dataset by utilizing the SMPL. AMASS [30] is an extensive human model parameter database that contains many human pose datasets. Specifically, we used the preprocessed data provided by Huang et al. [11]. The DIP-IMU dataset contains real IMU measurements and comes with preprocessed and raw data for each sequence, provided by Huang et al. We employed the raw data, filled the NaN values with the surrounding frame data, and turned the axis angle into a rotation matrix as the model output. The Total Capture dataset [18,19,20] also contains real IMU measurements. Following the IMU sensor settings of Yi et al. [12,13], the rotations and accelerations of the selected six IMUs were provided by the authors of the deep inertial poser (DIP) [11]. We used the DIP-IMU and Total Capture datasets as the evaluation data. Following the experimental procedure by Puchert et al. [14], for every model, we report values of a model trained only on synthetic data, as well as one fine-tuned on real data. Fine-tuning was performed on the subsets of the DIP-IMU and Total Capture datasets, and the test sets are separated from the training subsets. To test the model generalizability, the dataset was divided by subject. The DIP-IMU dataset has the corresponding subsets already assigned. The Total Capture dataset contains five subjects: the first three subjects were used to fine-tune the training, and the last two subjects were utilized as a test set.

#### 4.1.2. Evaluation Metrics

For a fair comparison, we employed the most common metrics to report the experimental results. The evaluation metrics are defined as follows:

(1) The DIP error is based on the DIP [11], which measures the error of the upper arm and thigh in the global coordinate system in degrees;
(2) The angle error represents the angle error of 15 body joints in the global coordinate system and is measured in degrees;
(3) The position error represents the three-dimensional position error of 15 important body joints and is measured in centimeters;
(4) The jitter error is based on the work of the Tanspose [12], which calculates the position of each joint to obtain the discrete value. This error is expressed as km/s^3^ and calculated as follows:

(13) $$J_{t}=\frac{p_{t}-3p_{t-1}+3p_{t-2}-p_{t-3}}{\Delta t^{3}},$$

where t denotes the current frame, p denotes the three-dimensional position of the node, and Δt denotes the time of each frame.

### 4.2. Contrast Experiment

For quantitative evaluation, we report the comparison of our models with the state-of-the-art methods based on sparse IMU pose estimation in Table 1, including the model trained only on synthetic data and the model fine-tuned on the training subsets partitioned by the DIP-IMU and Total Capture datasets. The experimental results demonstrate that the method proposed by Puchert et al. [14] performs better in the index of angle error. However, in terms of position error, the model in this paper achieves an optimal effect. The experimental results also demonstrate that the standard deviation of each index is the lowest for our model, indicating that it outperforms other state-of-the-art methods in processing more complex actions. Transpose [12] proposed by Yi et al. divides the human pose estimation task into three parts, and nearly every indicator exhibits significant improvement compared with DIP [11]. However, all tasks flattened the location features to model in time and did not consider the spatial location correlation, causing pose ambiguity. Therefore, we defined a unified framework for temporal and spatial modeling. The experimental results show that the proposed model is effective for the sparse IMU-based pose estimation task.

**Figure 6:**
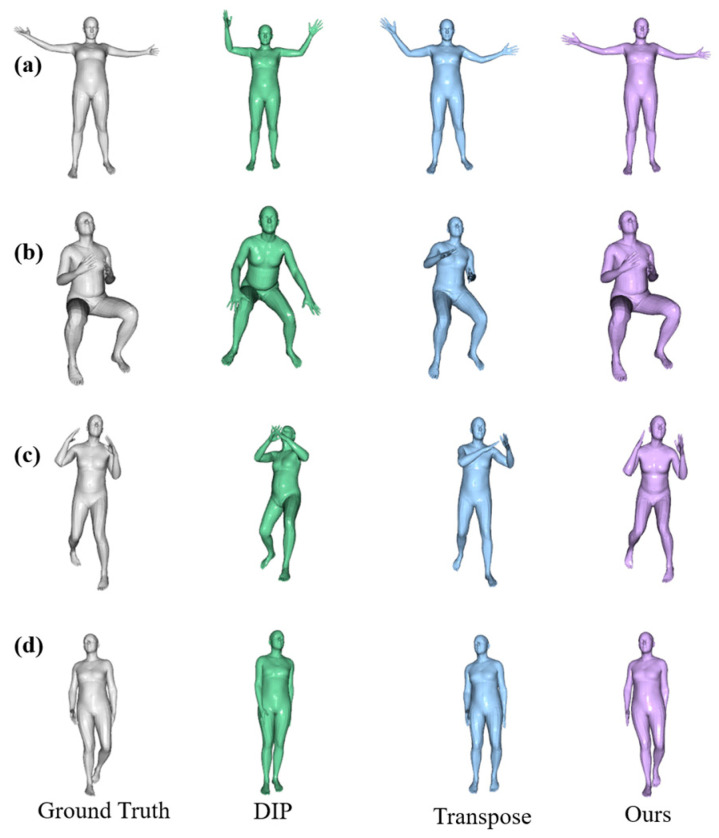
Qualitative results of our results compared to other methods on the DIP-IMU dataset. The results are “Ground Truth”, “DIP “, “Transpose” and “Ours” from left to right. Samples (**a**,**b**) show the reconstruction of the upper body by our network. The samples of (**c**,**d**) show the reconstruction of the lower body by our network.

For qualitative evaluation, we used the SMPL [15] for pose visualization, and the results are shown in Figure 6. As the networks predict only the parameters for the 15 core joints, those of the remaining eight joints for the hands and feet were set to identity. The root IMU measurements can directly convert the pose parameters of the pelvic joint, namely, the global body orientation. Figure 6 shows the visualization results of our proposed method compared to the ground truth and state-of-the-art methods on the DIP-IMU dataset. The case shown in the first two rows shows the reconstruction of the upper body by our method, the second row is the most ambiguous representative frame, and the cases shown in the third and fourth rows show the reconstruction effect of my method for the lower body. In the first presented example, we see that each network does not do the correct pose reconstruction, but our network is much better in fine details of the arm position. Our network can reconstruct pose with bent legs, as seen in the second row of the sitting. While aspects of the pose cannot be fully reproduced by the network, such as the arm and leg positions remaining poor, we note that the reconstruction is visually closer to the ground truth compared to DIP [11] and Transpose [12]. In the examples shown in the third and fourth, we see that while each network obtains this right in coarse structures, our network is much better at reconstructing fine details of leg positions, such as a straight and a slightly bent leg.

### 4.3. Ablation Experiment

This section analyzes the impact of the spatial attention network and the kinematic regression network. Table 2 presents the results of the ablation experiment conducted on our network model. We performed two groups of ablation experiments on the DIP-IMU dataset. The first group removed the spatial attention network and directly used linear regression, which is consistent with the kinematic tree structure, to generate the SMPL pose parameters, named “Only-regression”. The second group removed the spatial attention network and kinematic regression network and directly adopted multilayer perceptron (MLP) [31] to generate the pose parameters of the SMPL, named “MLP”. The experimental procedure was similar, and pre-training was performed on synthetic data. Fine-tuning was conducted using the training subset partitioned by the DIP-IMU dataset.

Based on the above experimental results, the spatial attention network has no significant effect on the jerk error. However, the accuracy of the other metrics significantly improved when the spatial attention network and kinematic regression network were combined. Learning the human joint relationship can significantly reduce the joint error when using only the human motion tree regression network, but combining the spatial attention mechanism with motion tree hierarchical regression produces better results.

Specifically, to verify the effect of the bottom-up regression process on the accuracy of each joint position, we test the joint position errors for two groups of ablation experiments, and the main results are shown in Table 3. In particular, the error of wrist and ankle joints reduce by 43.1% and 28.7%, respectively. Using the hierarchical relationships provided by human kinematics, the position errors of the nine key joints can be significantly reduced. The spatial attention network is used to pay more attention to the parent joint with more child joints.

## 5. Summary and Future Work

This paper proposed a temporal convolutional encoder and a human kinematics regression decoder to realize three-dimensional human pose estimation. This proposed regression network adopts the hierarchical relationship defined by the kinematic tree, with a spatial-attention network. This paper demonstrates that hierarchical regression based on temporal encoders and human kinematic topology can achieve better pose estimation for fast motion, as indicated by the following experimental results. (1) A reduction in error was achieved in both the cases of a model trained only on a synthetic dataset and a model fine-tuned on a dataset with actual IMU measurements. On the DIP-IMU dataset, the position error of the bone point was only 4.55 cm, approximately 19.4% less than that of the previous state-of-the-art methods. On the Total Capture dataset, the position error of the skeleton points was only 5.18 cm, approximately 13.6% less than that of the previous state-of-the-art methods. (2) Significant improvements in accuracy were achieved after the decoder was equipped with a spatial attention mechanism and human motion tree topology hierarchical regressor. Our model framework can be divided into three parts. In the early stage, the IMU measurements are encoded into high-dimensional features, and the time series feature difference of two consecutive frames is connected to generate 512-dimensional features. The middle stage is the extraction of temporal features at a deeper level of temporal coding. Simultaneously, the temporal convolutional layers gradually reduce the amount of computation. In the later stage, the relationship between joints is learned by relying on the human kinematics hierarchy. The input of the pose parameter regression network contains the local features output by the spatial attention network and the six-dimensional parameters of the relevant joints, and the regression generates the pose parameters. Based on the above methods, the encoder–decoder network framework was verified, and the temporal and spatial features were fused to enhance the robustness of sparse IMU-based human pose estimation.

Current applications include games, movie production, and virtual and augmented reality (VR/AR), which require real-time performance in daily working environments. Our method relies on sparse imu and can be applied to daily activities. Because the network’s pre-trained network model was trained on synthetic data and transfer learning was performed on the Total Capture and DIP-IMU datasets, the synthetic and real data are different from the experimental results, and it is necessary to understand the difference between the synthetic data and the actual measured data. In addition, our model relies on past and future frames and is an offline processing method. A subsequent consideration is whether online processing can be achieved. Finally, our method does not consider global human translation estimation, which can be added to the topics for future research.

## Figures and Tables

**Table 1:** Evaluation of our network model. Comparison with state-of-the-art and comparable methods on the DIP-IMU and Total Capture datasets. The evaluation metrics include the hip and shoulder global angle error (DIP Err) and the mean global angle, mean position, and mean jerk errors for all 15 key joints.

	Total Capture				DIP-IMU			
	DIP Err [°]	Ang Err [°]	Pos Err [cm]	Jerk Err $\left[km/s^{3}\right]$	DIP Err [°]	Ang Err [°]	Pos Err [cm]	Jerk Err $\left[km/s^{3}\right]$
Trained on synthetic data								
DIP [11]	30.00 (±19.09)	24.24 (±15.18)	14.87 (±9.33)	4.92 (±6.85)	33.17 (±19.16)	24.30 (±15.17)	13.74 (±8.29)	3.60 (±5.66)
Transpose [12]	18.49 (±15.68)	13.78 (±9.40)	8.22 (±6.82)	0.64 (±1.97)	29.92 (±16.78)	12.46 (±7.32)	8.10 (±6.08)	1.00 (±3.50)
Puchert et al. [14]	15.81 (±12.38)	12.53 (±8.41)	7.27 (±5.32)	1.16 (±2.61)	28.12 (±14.28)	11.35 (±6.28)	7.73 (±5.61)	1.12 (±3.55)
Ours	17.12 (±6.39)	15.09 (±5.46)	5.80 (±2.46)	1.06 (±1.38)	22.44 (±6.08)	19.73 (±5.77)	6.52 (±2.54)	1.12 (±1.41)
Fine-tuned on real-world data								
DIP [11]	17.45 (±15.59)	14.40 (±10.94)	8.26 (±7.26)	2.40 (±3.51)	17.75 (±11.77)	15.68 (±11.13)	7.71 (±5.43)	2.04 (±3.92)
Transpose [12]	17.03 (±14.74)	11.72 (±8.29)	7.43 (±5.95)	0.63 (±1.96)	18.52 (±13.50)	9.57 (±6.45)	6.71 (±4.95)	1.00 (±3.50)
Puchert et al. [14]	13.12 (±10.99)	10.12 (±7.03)	6.00 (±4.64)	1.08 (±2.46)	15.18 (±9.83)	8.13 (±5.23)	5.65 (±3.73)	1.13 (±3.54)
Ours	15.39 (±6.28)	13.37 (±5.35)	5.18 (±2.24)	0.74 (±0.93)	14.54 (±5.68)	13.76 (±5.21)	4.55 (±1.90)	1.02 (±1.31)

**Table 2:** Evaluation of the attention network and regression network for pose estimation on the DIP-IMU dataset. The evaluation is conducted by DIP, Angle, Position, and Jerk error metrics. The results of “Only-regression” and “MLP” are based on the settings described in the ablation study.

	DIP Err [°]	Ang Err [°]	Pos Err [cm]	Jerk Err $\left[km/s^{3}\right]$
Only-regression	15.14 (±5.33)	13.86 (±5.84)	4.71 (±1.82)	1.06 (±1.38)
MLP	36.46 (±15.32)	24.76 (±9.23)	5.98 (±2.30)	1.21 (±2.06)
Ours	14.54 (±5.68)	13.76 (±5.21)	4.55 (±1.90)	1.02 (±1.31)

**Table 3:** Evaluation of the position error of each joint in cm. Average distance error detection was performed on nine key joints of the human body on the DIP-IMU dataset.

Joint	Hip	Knee	Ankle	Shoulder	Elbow	Wrist	Back	Neck	Head
Only-regression	1.72	7.97	10.08	6.01	10.67	11.84	2.33	5.26	5.96
MLP	2.33	9.45	14.14	10.40	18.14	20.84	3.28	7.83	8.30
Ours	1.69	6.79	9.42	5.67	10.08	12.30	2.35	5.08	5.59

## Data Availability

The experiments are conducted on two public datasets, which are available at the following links: https://dip.is.tue.mpg.de/download.php.
